# Dip-Coating Self-Assembly Fabrication and Polarization Sensitive Photoresponse of Aligned Single-Walled Carbon Nanotube Film

**DOI:** 10.3390/s22020490

**Published:** 2022-01-10

**Authors:** Jiazhen Zhang, Luhan Yang, Huang Xu, Jie Zhou, Yuxiang Sang, Zhuangzhuang Cui, Changlong Liu, Jingjing Liu, Tianle Guo, Xingjun Wang, Lin Wang, Gang Chen, Xiaoshuang Chen

**Affiliations:** 1 State Key Laboratory of Infrared Physics, Shanghai Institute of Technical Physics, Chinese Academy of Sciences, Shanghai 200083, China; zhangjiazhen@mail.sitp.ac.cn (J.Z.); ylh134372543532021@163.com (L.Y.); 18621989862@163.com (H.X.); 1000478884@smail.shnu.edu.cn (J.Z.); sangyx1221@163.com (Y.S.); zh2cui@163.com (Z.C.); clliu@ucas.ac.cn (C.L.); liujingjing@mail.sitp.ac.cn (J.L.); guotianle@mail.sitp.ac.cn (T.G.); xjwang@mail.sitp.ac.cn (X.W.); wanglin@mail.sitp.ac.cn (L.W.); xschen@mail.sitp.ac.cn (X.C.); 2 University of Chinese Academy of Sciences, Beijing 100049, China; 3 School of Materials Science and Engineering, University of Shanghai for Science and Technology, Shanghai 200093, China; 4 Mathematics and Science College, Shanghai Normal University, Shanghai 200233, China

**Keywords:** aligned single-walled carbon nanotubes, self-assembly, aqueous suspension, dip-coating, negative photo response, polarimetry

## Abstract

It is challenging to obtain wafer-scaled aligned films for completely exploiting the promising properties of semiconducting single-walled carbon nanotubes (s-SWCNTs). Aligned s-SWCNTs with a large area can be obtained by combining water evaporation and slow withdrawal-induced self-assembly in a dip-coating process. Moreover, the tunability of deposition morphology parameters such as stripe width and spacing is examined. The polarized Raman results show that s-SWCNTs can be aligned in ±8.6°. The derived two terminal photodetector shows both a high negative responsivity of 41 A/W at 520 nm and high polarization sensitivity. Our results indicate that aligned films with a large area may be useful to electronics- and optoelectronics-related applications.

## 1. Introduction

Single-walled carbon nanotubes (SWCNTs) are possibly promising building blocks for next-generation electronics and optoelectronics applications such as high-performance thin-film transistors (TFTs), logic circuits [1,2], and infrared (IR) photodetectors [3,4], because of their ultra-fast charge transport mobility [5], compatible band gaps, remarkable electronic properties, great mechanical and chemical stabilities [3,4], and good compatibility with complementary metal-oxide semiconductor (CMOS) fabrication processing. However, to exploit the exceptional properties of SWCNTs, one of the most difficult challenges is to assemble highly enriched s-SWCNT arrays with better alignment [6,7,8].

To date, direct growth and solution-based assembly are two important methods to obtain aligned carbon nanotube (CNT) arrays [8]. In the former method, chemical vapor deposition (CVD) is the most promising approach because of controlled growth and inexpensive fabrication [6]. Nevertheless, there is room for high s-SWCNT density and purity for future applications. Moreover, this method usually requires a temperature of >800 °C [8]; therefore, it is currently incompatible with organic or flexible electronics. Due to the synergistic contribution of solution-based purifying techniques by type, diameter, and chirality using density gradient ultracentrifugation [9], column chromatography [10], and aqueous two-phase extraction [11], the solution-based assembly method has drawn considerable attention because it is easy, inexpensive, and able to cover a large area at room temperature [8,12]. Dip-coating, a typical solution-based assembly approach, is quite simple [13] in processibility, efficient in fabrication [14], scalable [15] for large and non-planar substrates [13,16], and reproducible for mass production [15,16].

Previous studies (Table 1) have primarily focused on a mono-phasic dip-coating method where a monophasic liquid, aqueous solution [17,18,19,20,21], or organic solution [13,14,15,16,22,23] is used to suspend CNTs. To date, it is difficult to fabricate aligned CNT arrays. Based on these studies, a random network rather than an aligned array occurs, possibly because of the following reasons: (1) strong attraction of functionalized substrate surface catches suspended CNTs before nanotubes are oriented to a dominant direction [24]; (2) fast solvent volatilization deposits CNTs early before their ordered orientation [23]; and (3) CNTs in large aspect ratios are not sufficiently stiff and are easy to be entangled and curved [25]. Recently, there have been certain studies on bi-phasic dip-coating techniques [12,23,26] to fabricate full coverage and highly aligned thin films [12,23]. On the one hand, Joo et al. [26] and Liu et al. [12] use a small volume of an easily volatile organic solvent to suspend and confine CNTs in a thin layer on another immiscible liquids. On the other hand, Gao et al. [23] uses chloroform to suspend CNTs at the top of which a thick layer of DI water is used to seal the whole suspension and limit volatilization.

In this work, a slow and mono-phasic dip coating approach is used to prepare large-scale, highly aligned, and periodic stripe-like thin films. In terms of the reasons failing to fabricate aligned arrays, we select slow withdrawal speeds, rigid SWCNTs, and nonvolatile deionized (DI) water as solvents to guarantee an ordered deposition. Unlike recent bi-phasic studies, this method is simpler in processibility and avoids the usage of toxic reagents. Both scanning electron microscopy (SEM) and polarized Raman spectra show that SWCNTs deposit in a high alignment. For deposition dimensions, the stripe width is independently controlled using the withdrawal speed and SWCNT concentration; however, they have limited controllability on stripe spacing. Subsequently, the as-prepared film is fabricated as a symmetrical two-terminal photodetector. Under the illumination of a wavelength of 520 nm, the device shows a high photoresponse and polarization sensitivity. Subsequently, a linear polarizer is used to shade the sample and tune the angle between the polarization direction of light and film strip direction. We identify photocurrents against the angle that is in a good fitting of the cos^2^ relation. The derived high ${\left(I_{photo}\right)}_{max}/{\left(I_{photo}\right)}_{min}$ reconfirms the high array alignment of the dip-coating self-assembly film.

## 2. Materials and Methods

### 2.1. Suspension Preparation

All aqueous SWCNT suspensions were commercially purchased, and then they were added to two different anionic surfactants, sodium dodecyl sulfate (SDS) and sodium deoxycholate (DOC), respectively, to ensure the suspensions were stable. As per SWCNTs with dispersant and different semiconducting purity (unsorted or purified to >99%), the dispersions could be categorized as follows: P1 type (arc-discharge unsorted nanotubes dispersed with SDS), P2 type (arc-discharge unsorted nanotubes with DOC), and P3 type (CVD-grown TUBALL purified nanotubes with SDS). Initially, all suspensions shared the same colloid concentration of ~10 mg/mL and surfactant concentration (~1 wt.%). After sonication and centrifugation, the SWCNT concentrations reduced to 0.419 (P1), 0.488 (P2), and 0.700 mg/mL (P3), respectively. The stock solutions were then diluted with deionized (DI) water to a desired concentration, and then sonicated in a bath at 40% amplitude for 30 min before use.

### 2.2. SWCNT Film Deposition

Dip coating was conducted on an optical table to avoid any possible vibrations in ambient conditions (~17 °C, RH = 50–70%). A micrometer programmable dip-coater (SYDC-100) was used to vertically hold both wafers and cut square substrates, and then lift upward at a prescribed speed (0.10, 0.15 and 0.20 μm/s) above a plastic cuvette (1.0 × 1.0 × 4.5 cm^3^) filled with a diluted colloidal solution after the substrate was dipped in the solution. Prior to coating, Si/SiO_2_ substrates were successively ultrasonicated for 15 min with acetone and ethanol, followed by a DI water washing and nitrogen drying. Subsequently, substrates were treated in oxygen plasma (100 W, 90 s) to make it solventphilic. Finally, as-deposited films were extensively rinsed with DI water to remove excess surfactants.

### 2.3. Device Fabrication

For the photodetector, the films derived were first transferred on highly resistant Si (ρ ≈ 20,000 Ω cm) capped with a thin oxide layer SiO_2_ (300 nm). Symmetrical two-terminal photodetector were fabricated by depositing Cr (15 nm)/Au (45 nm) metal electrodes on the coated s-SWCNT enriched (purity > 99%) film with a UV lithography process and thermal evaporation. Then, a second UV-lithography step, followed by oxygen plasma etching, was used to define and separate devices with a channel length of 10 μm and a channel width of 40 μm.

### 2.4. Characterization

SEM images were obtained using a JEOL JSM-7800F field-emission scanning electron microscope at 5 kV and 10.3 mm WD to examine film morphology. Raman spectra were obtained using a RAMHR 800 system from Horiba Jobin Yvon Lab with a laser excitation wavelength of 532 nm. Polarized micro-Raman spectroscopy was obtained in a quasi-back-scattering geometry using ~20 mW at the 514.5-nanometer line of Ar-ion laser equipped with an objective of 100× as the excitation source. All electrical and opto-electrical measurements were performed in air using Keysight B2912A. The measurements of anisotropic photocurrent versus light polarization were characterized by introducing a linear polarizing filter between the sample and incident beam laser.

## 3. Results and Discussion

### 3.1. Ordering Mechanisms and Deposition Geometry

**Figure 1:**
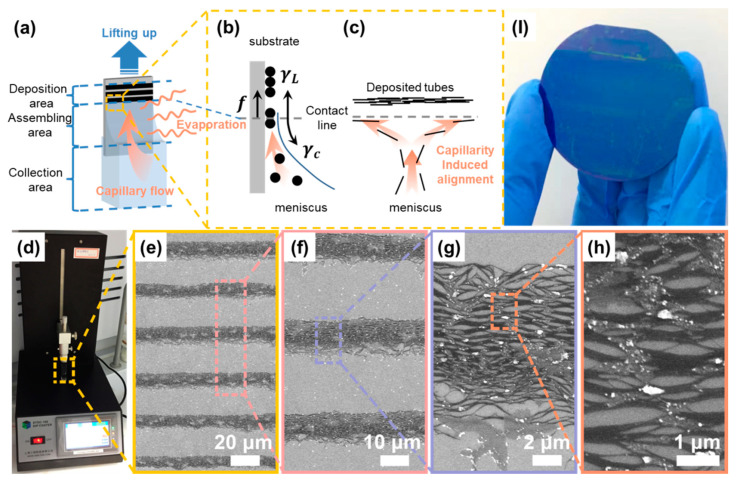
The dip-coating self-assembly fabrication of aligned SWCNT film. (**a**) Schematic of the coating process; (**b**) The deposition process driven by solvent evaporation; (**c**) The aligning process induced by capillary flow; (**d**) The experimental setup for the dip-coating processing; (**e**–**h**) SEM images for the deposited SWCNTs in different resolution (The regions in dark/light color refer to SWCNTs/substrate) and (**I**) as-deposited SWCNT film on a 2-inch wafer.

As shown in Figure 1a, a Si/SiO_2_ substrate is once dipped in a container (200-milliliter glass beaker for a 2-inch wafer substrate; 4.5-milliliter cuvette for a 0.8 × 1.2 cm^2^ substrate) filled with an aqueous SWCNT suspension, and then the liquid solvent attaches to the substrate owing to the intermolecular forces between the solvent and the -OH groups on a hydrophilized substrate surface [27]. The capillary effect driven by water evaporation pulls the bulk liquid up, and then forms a concave meniscus because of the interfacial energy at the solid–liquid–vapor contact line [16]. We conducted the same dip-coating process to hydrophobic substrates such as Si. It had been reported to have a sparsely dot-like and ordered stripe-like pattern with and without hydrophilization treatment (substrate immersed in a Piranha solution [16,27] (H_2_SO_4_:H_2_O_2_, volume ratio of 3:1) for ~1 h). The images are not shown here, and the phenomenon confirms that the wettability of the substrate is important for the self-assembly process [28,29]. Furthermore, wettability affects the curvature of the meniscus [27]. As per Núñez et al.’s observation [27], three areas can be defined based on the dynamic assembly of particles, i.e., (i) the deposition area, (ii) assembling area, and (iii) collection area, as shown in Figure 1a. The deposition area is a region in which only sediments remain after the solvent entirely evaporated. In the assembling area or meniscus tip, particles are densely packed and embedded in a thin-film solvent. Finally, in the collection area or the rest of meniscus, the particle concentration is higher than that of the bulk suspension because of both the continuous particle influx carried by a capillary flow [16,30] and the larger evaporative solvent loss at the top of the meniscus with respect to the remaining part [31].

To summarize, using dip-coating to coat ordered films is a solvent evaporation-induced self-assembly process that involves particles transported by a convective flow [29,30,32,33,34] with particles depositing at the meniscus tip because of supersaturation [35,36]. Although the mechanism of stripe-like film formation is not entirely understood to date, a generally accepted explanation is that the pinning [31,37,38] of the contact line caused either by the surface irregularities of substrates [32,39] or by self-pinning between suspended particles with the substrate, is a prerequisite for the formation. Engel et al. [39] provided a “stick–slip” model for aqueous SWCNT suspension to describe the pinning/de-pinning motion of the contact line. As shown in Figure 1b, contact line pinning and de-pinning are in competition between pinning (frictional force, f, and liquid surface tension, $\gamma_{L}$) and de-pinning forces (capillary force, $\gamma_{c}$) [30,32,39]. On the one hand, when the former proportion is dominant, the contact line arrests on the substrate surface. For a sessile droplet on a flat substrate, the evaporative vapor flux diverges at a contact line as $\left|\vec{j}\right|\propto r^{-\left(\pi -2\theta \right)/\left(2\pi -2\theta \right)}$ [31], where r is the distance from contact line, and θ is the contact angle. The divergence is possibly similar for this entrained and elongated meniscus on a vertical substrate; thus, at the contact line, the higher evaporation rate [30] generates an outward capillary flow to pin the contact line by drawing a solvent from the liquid bulk [31], which carries dispersed particles in the assembling area edge. The evaporative loss of solvent along with particle influx contributes to supersaturation, and then precipitates out as nuclei at the assembling area [36]. On the other hand, when the de-pinning force dominates, the contact line becomes unstable and slips down to a new position until the reshaped meniscus subsequently pins it [36]. Eventually, the repetitive pinning/de-pinning motion results in a striped film pattern. Finally, Li et al. [40] proposed another “pinning-zipping” model and revealed that kink propagation rather than the slippage of contact line facilitates an advancement from de-pinning to pinning using an in situ optical observation. Although the particle deposition mechanism is unclear, the abovementioned models provide clues to the stripe-like film formation mechanism.

In the assembly area, concentrated SWCNTs with a high aspect ratio favor a lateral alignment previously explained by Onsager on the basis of the excluded volume entropy model [41]. Furthermore, to intrinsically disperse insolvable SWCNTs, surfactants are used to wrap and stably disperse nanotubes and provide an inter-tube steric effect or even electrostatic repulsion, which can then inhibit the agglomeration in the aligned SWCNT film formation process [23]. Moreover, both hydrodynamic forces [40], such as the capillary force and the effect of geometrical constraint [42] close to the contact line, possibly improve the ordering and aligning of nanotubes along the contact line in the lowest energy [32] and sterically favored [26] configuration (Figure 1c).

Figure 1d shows the dip-coating apparatus for preparing ordered SWCNT arrays on a 0.8 × 1.2 cm^2^ Si/SiO_2_ substrate. A number of magnified scanning electron microscopy (SEM) images (Figure 1e–h) of the arrays are used to demonstrate the details, particularly a good alignment across a hundred micrometers where dark stripes are SWCNTs, and grey stripes are spacings between two adjacent SWCNT stripes. Note that white impurities on the film are residual surfactants only after being rinsed with DI water a few times. Moreover, we performed the dip-coating process to coat ordered arrays onto a 2-inch wafer with the SWCNT coverage region marked in blue (Figure 1I). This indicated that the method is universal for large-sized substrates and possibly for arrayed electronics and opto-electronics.

### 3.2. Surfactant Effect

**Figure 2:**
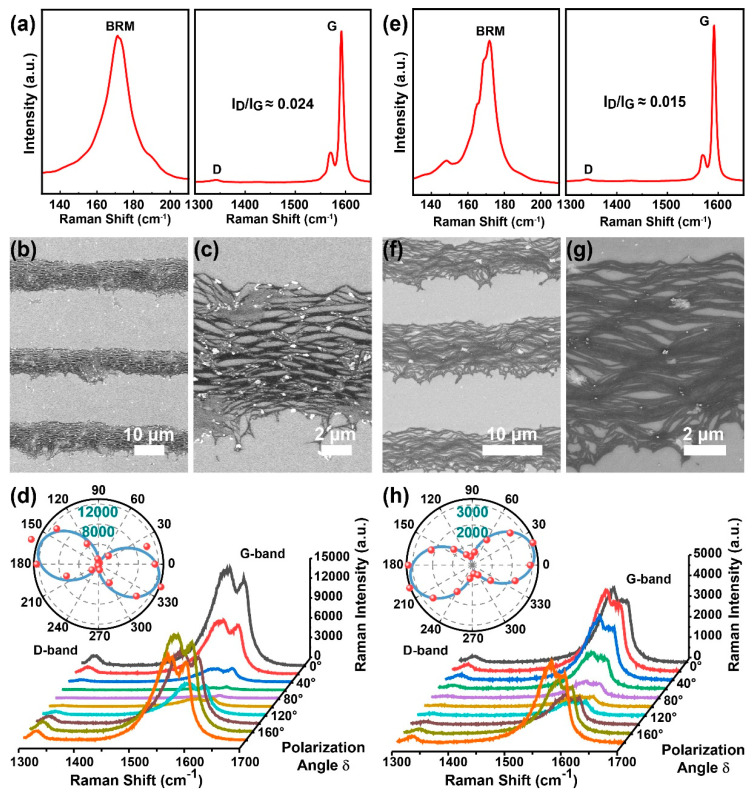
The corresponding Raman, SEM images, and polarized Raman patterns for SWCNT films prepared using SDS (**a**–**d**) and DOC (**e**–**h**) surface surfactants.

To obtain highly aligned SWCNT films, we conducted the dip-coating process to two suspensions (types P1 and P2) with different but extensively used anionic surfactants (SDS and DOC) because these amphiphilic molecules have considerably different adhesive interactions on SWCNTs for multiple molecular structures. The dispersed SWCNTs share a similar aspect ratio, namely, a similarly averaged nanotube length of ~1–2 μm (see Figure S1) and diameter of ~1.3–1.7 nm (see Figure 2a,e and Figure S2) derived from an empirical formula ω=248/d [43,44], where ω is the peak position of the radial breathing mode (RBM) and d is the diameter. As shown in Figure 2a,e, for both as-deposited nanotubes, the BRM, D, and G bands peak at the same Raman shift of ~171 ± 7, 1342, and 1590 cm^−1^, respectively. Furthermore, the SWCNT impurities, including defects in the graphene sheet and graphite-like carbons [14], can be characterized using the D/G band intensity ratio [45]. Comparable intensity ratios of D and G bands (0.024 and 0.015) for these samples show nearly the same and a small number of defective structures in SWCNTs.

Figure 2b,c,f,g show the high- and low-resolution SEM images of as-deposited SWCNT (types P1 and P2) stripes in randomly selected regions, excluding places close to the substrate edge to avoid the boundary effect [23], as well as a concave shape and convergent stripes. The concave-shaped stripes are possibly caused by an observably concaved meniscus because of different interfacial energies between the Si/SiO_2_ substrate and polycarbonate container walls. Both groups of SEM images show a high degree of alignment. They are then quantified via polarized Raman spectra. Figure 2d,h show spectra in the vicinity of the G band from 1300 to 1700 cm^−1^ as a function of angle, φ, between the polarization of the Raman excitation laser and the stripe long axis. The G band intensity versus φ is plotted in polar coordinates (insets in Figure 2d,h), indicating an intensity ratio of 44.6 and 11.4 between the maximum Raman intensity ($I_{max}$) and the minimum Raman intensity ($I_{min}$), respectively.

We assume the orientation of as-prepared SWCNTs in the stripes obeys a Gaussian angular distribution [12,26]. The probability, f(δ), of identifying an SWCNT with its long axis misaligned from the stripe direction by the angle δ is as follows:

(1) $$f\left(\delta \right)=exp\left(\frac{-\delta^{2}}{2\sigma^{2}}\right)$$

where σ is the width of the angular distribution. Moreover, as previously reported, the G band intensity follows a cos^2^ dependence with the Raman excitation laser polarization [46,47]; thus, the ratio of the maximum G band intensity to the minimum G band intensity for excitation polarized parallel versus normal to the stripe is as follows [48]:

(2) $$\frac{I_{G}\left(\varphi =0{}^{\circ}\right)}{I_{G}\left(\varphi =90{}^{\circ}\right)}=\frac{\int_{-\pi /2}^{\pi /2}f\left(\delta \right)cos^{2}\left(\delta \right)d\delta }{\int_{-\pi /2}^{\pi /2}f\left(\delta \right)sin^{2}\left(\delta \right)d\delta }$$

The intensity ratio of 44.6 indicates that the alignment between P1-SWCNTs in the stripe is in ±8.6° and better than that of P2-SWCNTs. The higher alignment may be attributed to more appropriate inter-tube interactions, including electrostatic repulsion, because of wrapped SDS molecules. Thereafter, we selected SDS to disperse SWCNTs to obtain a highly aligned film rather than DOC.

### 3.3. Deposition Controllability

**Figure 3:**
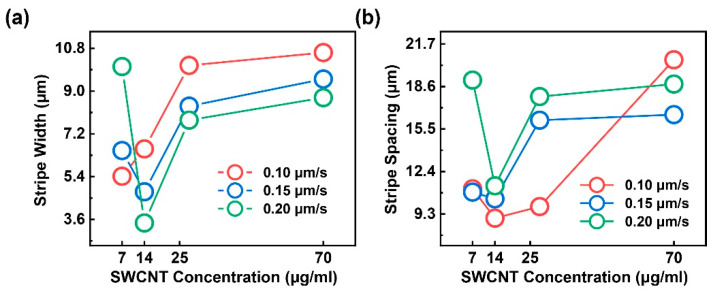
(**a**) shows the SWCNT stripes width as functions of the concentration and withdraw speed; (**b**) is the SWCNT stripe spacing as functions of the concentration and withdraw speed.

**Figure 4:**
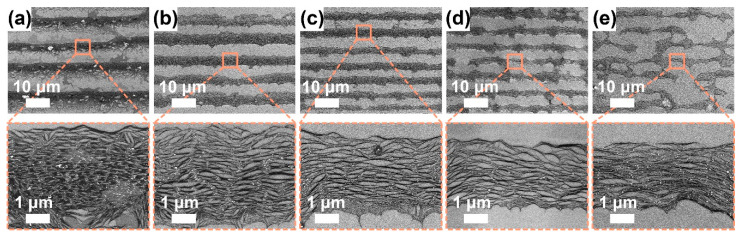
SEM images for P3-SWCNT films at a fixed SDS concentration (~1.0 mg/mL), withdrawal speed (0.1 μm/s), environment temperature (~17 °C) and humidity (~50–70%), but varied SWCNT concentrations: (**a**) ~70.0, (**b**) ~25.0, (**c**) ~14.0, (**d**) ~9.3, and (**e**) ~7.0 μg/mL. To characterize the alignment of SWCNTs in stripes, high-resolution SEM images were shown below.

To examine the effects of the SWCNT concentration and withdrawal speed on stripe morphology, we performed the slow dip-coating process to P3-SWCNTs dispersions at various concentrations (7–70 μg/mL) and withdrawal speeds (0.10–0.20 μm/s) in ambient air. The averaged stripe widths and spacings are measured from the SEM images. As shown in Figure 3a, an increase in nanotube concentration generally leads to an increase in wider stripes. This is qualitatively natural because, for a certain convective flow, additional particles can be carried to the meniscus tip at the assembling area, and thus result in wider [32,40,49] and usually thicker [29,33] stripes. The result can be explained using the “stick–slip” model [32]. In particular, a higher SWCNT concentration contributes to a larger number of particles at the contact line; thus, the frictional force (f) grows stronger and requires a larger capillary force ($\gamma_{c}$) to depin the contact line while liquid surface tension ($\gamma_{L}$) is nearly constant, and then wider stripes are generated. In terms of the withdrawing effect, the stripe width is almost inversely proportional to the withdrawal velocity, as reported by Watanabe et al. [49]. Under this circumstance, the withdrawing counteracts the capillary flow and results in a reduction in the number of particles transported to the assembling area because both components are in an upward motion. Consequently, a relatively weak particle influx close to the contact line leads to a smaller pinning force. Therefore, de-pinning easily occurs, and then narrower stripes are generated. Note that the similar and increasing trends of all the stripe widths versus the SWCNT concentration curves at different withdrawal rates may indicate an independent relation between concentrations and withdrawal speeds. An irregularity at a low SWCNT concentration may be attributed to degraded ordering and a possible concentration-induced deposition morphology transition from stripe-like, to punch-hole-like, to dot-like (Figure 4d,e) [35,40].

In Figure 3b, the stripe spacings are ambiguously proportional to the SWCNT concentration at a specific withdrawal rate of 0.10, 0.15, and 0.20 μm/s. The irregularity at a low SWCNT concentration of 7 μg/mL may be attributed to degraded ordering and a possible deposition geometry transition. On the one hand, according to Engel’s report [39], on the basis of the “stick–slip” model, the spacing between successive rows of CNTs is considered to be pinning possibility dependent, namely, solution concentration-dependent in an evaporation-induced self-assembly process. Consequently, spacing is expected to be inversely proportional to SWCNT concentration. On the other hand, Watanabe et al. [49] and Mino et al. [33] propose another qualitatively and quantitively valid model. It is considered that stripe spacing forms when the concave meniscus surface with its top edge attached to the outermost particle layer is curved and then adheres to the substrate. A rate difference between stripe growth and evaporation rates contributes to the curvature. Watanabe’s conclusion, i.e., the stripe spacing seems not to directly depend on the solution concentration but on the number of layers of stripes, may show a multi-layered deposition in this study; the film thickness is a function of solution concentration. At multiple withdrawal speeds, the withdrawing is extremely slow, such that the meniscus shape is maintained, as described by the Laplace equation in static mechanics without being entrained via an upward moving substrate [49]. Therefore, as shown in Figure 3b, the withdrawal may provide a limited control on the deposition dimensions.

#### 3.3.1. Solution Concentration Effect

To investigate the SWCNT concentration effect, we perform the dip-coating process to P3-SWCNTs at multiple SWCNT concentrations when maintaining a fixed withdrawal speed of 0.1 μm/s because the withdrawal speed is predicted to be independent of the SWCNT concentration. In the low-resolution SEM images (Figure 4a–e), an SWCNT concentration-dependent deposition morphology transits from a stripe-like to punch-hole-like pattern with fingering instabilities [35,50]. Eventually, the liquid fingers deposit in bumps perpendicular to stripes, similar to the famous “tears of wine”, because of competition among capillary, viscous and Marangoni forces [50]. When the concentration reduces to 7 μg/mL, the stripes grow discontinuous and narrow to a few micrometers while the bumps grow wide. A dot-like deposition is reasonably predictable if the concentration continues to decrease. At high solution concentrations, a parallel superlattice structure is formed and originates from the repetitive “stick-slip” motion of the contact line. Note that when the concentration increases to 70 μg/mL, SWCNT clusters occur at the stripe lower edges and unexpectedly orient in a normal direction to the stripe direction. Li et al. proposed that the alignment is attributed to the capillary flow significantly enhanced by the continuous pinning of the contact line and accelerated solvent evaporation [42]. However, because films were fabricated at the same temperature, the explanation may be not sufficient for the condition. At low concentrations, the stripes become wavy, possibly because a small number of SWCNTs on the meniscus tip cannot pin the contact line firmly enough [33]. In the high-resolution SEM images (Figure 4a–e), most SWCNTs in the stripes visually stay highly aligned, even at low concentrations.

#### 3.3.2. Withdrawing Effect

**Figure 5:**
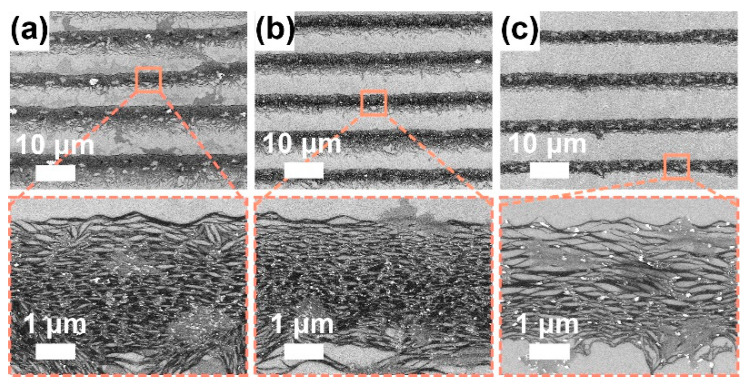
SEM images for P3-SWCNT films at a fixed SWCNT concentration (~70 μg/mL), SDS concentration (~1.0 mg/mL), environment temperature (~17 °C) and humidity (~50–70%) but varied withdrawal speed: (**a**) 0.10, (**b**) 0.15, and (**c**) 0.20 μm/s. To characterize the alignment of SWCNTs in stripes, high-resolution SEM images were observed.

Subsequently, for the P3-SWCNT solution at a fixed concentration of 70 μg/mL, we adopt three different withdrawal speed of 0.10, 0.15, and 0.20 μm/s, to study the withdrawing effect. In the low-resolution SEM images (Figure 5a–c), the perpendicularly oriented clusters gradually disappear as the withdrawal speed increases to 0.20 μm/s. Therefore, it may be a withdrawal speed-related phenomenon involved with the capillary flow, as reported by Li et al. [42]. In the high-resolution SEM images (Figure 5a–c), the alignment in the stripe seems to remain, and the film thickness seems to decrease because faster withdrawing allows fewer particles to be transported to the assembling area and results in thinner stripes [49]. However, as shown in Figure S3, the fast withdrawing (0.20 μm/s) seems to deposit in a discrete stripe-like pattern with great degradation in ordering degree, when the SWCNT concentration becomes moderate (~14 μg/mL). Eventually, fast withdrawal (0.20 μm/s) allows the deposition duration to accelerate from a few days (at a zero-withdrawal speed) to ~10 h for a 1.0-cm-long substrate.

### 3.4. Polarization Sensitive Photodetector

**Figure 6:**
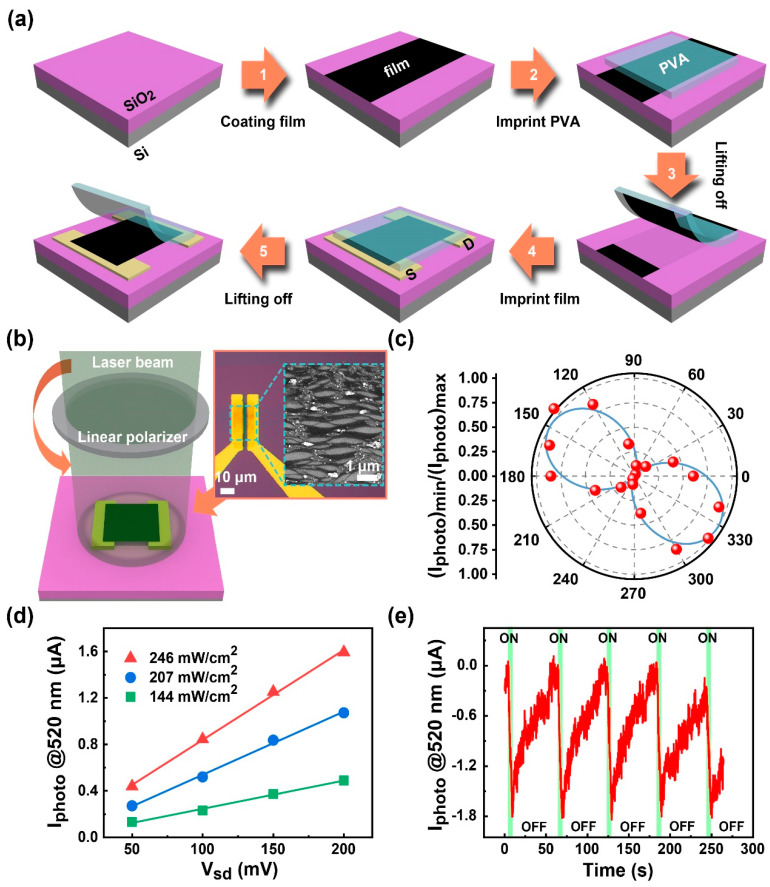
(**a**) A regular 2D film transferring process applied to the SWCNT film; (**b**) Schematic of photoresponse measurement for a two-terminal device; (**c**) Polarization-dependent photocurrent observed for aligned SWCNTs; (**d**) Photocurrent as a function of bias voltage under different incident power; and (**e**) Temporal photoresponse of $I_{photo}$ measured under $V_{sd}$ of 200 mV.

To characterize the anisotropy and alignment of as-prepared films, two terminal photodetectors for photoresponse testing are applied. In the device fabrication process (Figure 6a), films are rinsed, dried, and then transferred on a pair of pre-patterned symmetrical electrodes with SWCNTs aligned parallel to the channel (Figure 6b, inset). As shown in Figure 6b, under the polarized light of illumination, photocurrents versus rotation angles are obtained. Figure 6c shows that the device is polarization-sensitive, and the measured polarization ratio of the photocurrent magnitude ${\left(I_{photo}\right)}_{max}/{\left(I_{photo}\right)}_{min}$ is 113.52. The value is larger than a newly reported magnitude for an aligned SWCNT photodetector [51]. Figure 6d shows the photoelectronic performances under different illumination power intensities. The photocurrent gradually increases as the optical power increases from 144 to 246 mW/cm^2^. The linear relation between the photocurrent and the bias voltage shows a typical photoconductive effect [52]. Figure 6e shows the on/off source–drain current of the photodetector at an incident power density of 207 mW/cm^2^ (520 nm, *V_sd_* = 200 mV). The photocurrent dramatically decreases on illumination, and then slowly recovers in the dark. The negative photocurrent may be attributed to the oxygen molecule adsorption [53,54,55]. The pristine SWCNTs are usually p-type doped because of defects [51] and the exposure in the air [56,57]. In the dark, adsorbed O_2_ molecules behave as electron acceptors [58]; thus, they capture free electrons and result in increased electrical conductivity. Under the illumination, photons (2.39 eV) generate electron-hole pairs. Photoinduced holes firstly recombine with trapped electrons due to a low binding energy between O_2_ and SWCNTs (~0.25 eV) [58] in the O_2_ photodesorption process. Free photoinduced electrons subsequently recombine with the majority of carriers and lead to decreased electrical conductivity. Besides the oxygen adsorption/photodesorption, other thermal and nonthermal processes may also contribute to the NPC phenomenon. Although CNT involving NPC has been studied early [59], the research is still in its infancy [60]. Therefore, further exploration is still needed in the future. In addition, when the time of the “light on” state is sufficiently prolonged, the $I_{photo}$ firstly descends sharply, then approaches saturation. When the time of the “light off” state is long enough, the $I_{photo}$ climbs slowly and stays in a plateau pattern. The anomalous photoresponse, the majority of carriers contributing to the photocurrents, has the potential to provide a way for the high-sensitivity room-temperature photodetection [61].

The responsivity of a photodetector can be defined as $R=I_{photo}/PA$, where $I_{photo}$ is the photocurrent, P is the incident power, and A is the effective area of the detector. The derived responsivity *R* is ~41 A/W at 520 nm.

## 4. Conclusions and Future Work

In this study, we reported a simple and scalable approach using a mono-phasic dip-coating method at a low withdrawal speed to produce highly ordered SWCNT films in a parallel stripe pattern. The coating morphology parameters such as stripe width and spacing were tunable with a colloidal concentration and withdrawal rate, respectively. Both the optical and opto-electrical measurements demonstrated a high degree of alignment in the films. The approach may contribute to ordering and depositing mechanisms in the dip-coating process. Note that additional improvements for deposition in small bundles and uniformity in inter-tube pitch and film thickness were promising for high-performance TFTs, ICs, and IR photodetectors, including flexible electronics.

## Figures and Tables

**Table 1:** Comparison of Various Dip-coating Approaches for Aqueous Suspension. (M: mono-phasic; B: bi-phasic).

Materials	Solvent (Configuration)	Withdrawal Speed	Alignment	Ref.
SWCNT	DI water (M)	50 mm/min	random	[17]
	DI water (M)		random	[19]
	DI water (M)	0.5 cm/min	random	[21]
	DI water (M)		random	[20]
	DI water (M)	0.1–0.2 μm/s	aligned	this work
	1,2-DCB (M)	0.3–6 mm/min	random	[16]
	CSA (M)		random	[13]
	DCE (M)	50 mm/min	random	[14]
	DI water/chloroform (B)	200–1000 μm/s	aligned	[23]
	C_4_H_8_O_2_/chloroform (B)		aligned	[12]
	chloroform/DI water (B)	5 mm/min	aligned	[26]
SWCNT (DWCNT)	CSA (M)	1–3 mm/min	random	[15]
DWCNT	CSA (M)	0.5–10 mm/min	random	[22]
CNT	D_2_O (M)	50–2000 nm/s	random	[18]

## Data Availability

Not applicable.

## Supplementary Materials

The following supporting information can be downloaded at: https://www.mdpi.com/article/10.3390/s22020490/s1, Figure S1: SEM images for P1-, P2-, and P3-SWCNTs; Figure S2: Raman spectrum for as-prepared P3-SWCNT film added with SDS; Figure S3: SEM images to illustrate the withdrawal speed effect for P3-SWCNT films at different withdrawal speeds.
