# Supplementary material for: Dip-Coating Self-Assembly Fabrication and Polarization Sensitive Photoresponse of Aligned Single-Walled Carbon Nanotube Film
Source: Sensors (Basel). 2022 Jan 10;22(2):490. doi: 10.3390/s22020490 (PMC8779663; doi:10.3390/s22020490)
Supplement: Supplementary file 1 [file sensors-22-00490-s001.zip › sensors-1510309-supplementary.pdf]

Supporting Information

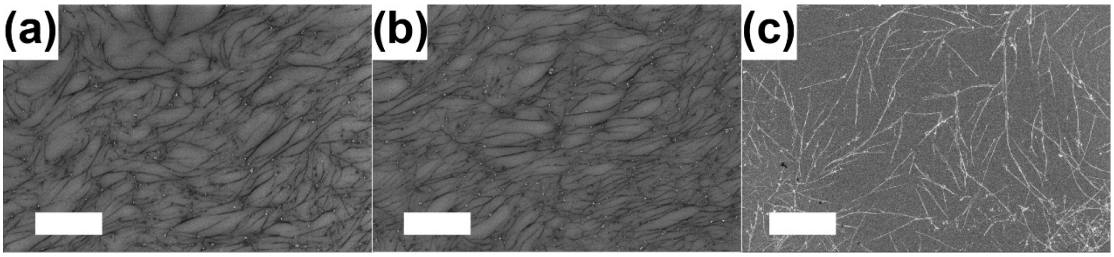

Figure S1. SEM images (scale bar, 2 μm) for P1-, P2-, and P3-SWCNTs.

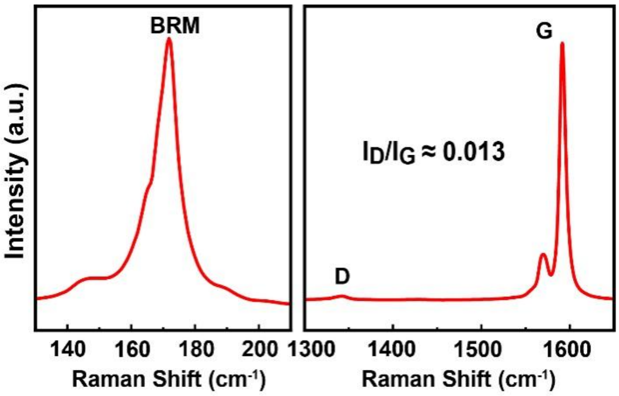

Figure S2. Raman spectrum for as-prepared P3-SWCNT film added with SDS.

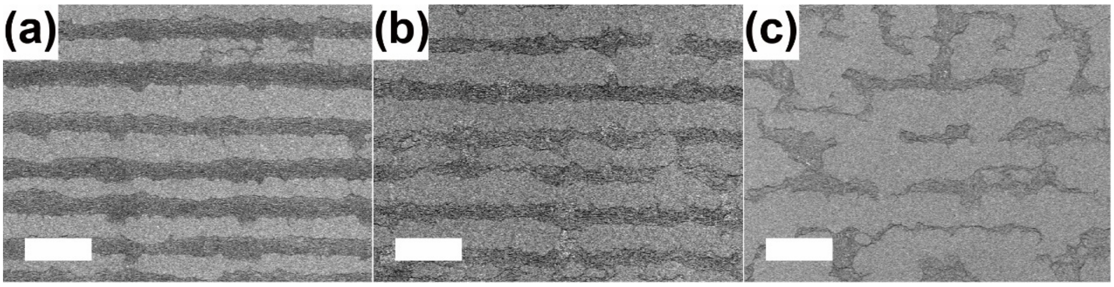

Figure S3. SEM images (scale bar, 10 μm) to illustrate the withdrawal speed effect for P3-SWCNT films at different withdrawal speeds (0.10, 0.15 and 0.20 μm/s), and with a fixed SWCNT concentration (~14 μg/ml) and under ambient conditions (~17 ℃, RH=~50-70%).
